# Diagnosing onset of labor: a systematic review of definitions in the research literature

**DOI:** 10.1186/s12884-016-0857-4

**Published:** 2016-04-02

**Authors:** Gillian E. Hanley, Sarah Munro, Devon Greyson, Mechthild M. Gross, Vanora Hundley, Helen Spiby, Patricia A. Janssen

**Affiliations:** Department of Obstetrics & Gynaecology, University of British Columbia, Vancouver, BC Canada; Interdisciplinary Studies Department, University of British Columbia, Vancouver, BC Canada; Midwifery Research and Education Unit, Hannover Medical School, Hanover, Germany; Faculty of Health & Social Sciences, Bournemouth University, Bournemouth, UK; School of Health Sciences, University of Nottingham, Nottingham, UK; School of Population and Public Health, University of British Columbia, Vancouver, BC Canada; Child and Family Research Institute, University of British Columbia, Vancouver, BC Canada

## Abstract

**Background:**

The diagnosis of labor onset has been described as one of the most important judgments in maternity care. There is compelling evidence that the duration of both latent and active phase labor are clinically important and require consistent approaches to measurement. In order to measure the duration of labor phases systematically, we need standard definitions of their onset. We reviewed the literature to examine definitions of labor onset and the evidentiary basis provided for these definitions.

**Methods:**

Five electronic databases were searched using predefined search terms. We included English, French and German language studies published between January 1978 and March 2014 defining the onset of latent labor and/or active labor in a population of healthy women with term births. Studies focusing exclusively on induced labor were excluded.

**Results:**

We included 62 studies. Four ‘types’ of labor onset were defined: latent phase, active phase, first stage and unspecified. Labor onset was most commonly defined through the presence of regular painful contractions (71 % of studies) and/or some measure of cervical dilatation (68 % of studies). However, there was considerable discrepancy about what constituted onset of labor even within ‘type’ of labor onset. The majority of studies did not provide evidentiary support for their choice of definition of labor onset.

**Conclusions:**

There is little consensus regarding definitions of labor onset in the research literature. In order to avoid misdiagnosis of the onset of labor and identify departures from normal labor trajectories, a consistent and measurable definition of labor onset for each phase and stage is essential. In choosing standard definitions, the consequences of their use on rates of maternal and fetal morbidity must also be examined.

## Background

The diagnosis of labor onset has been described as one of the most difficult and important judgments made by providers of maternity care [1]. The first stage of labor, through effective uterine contractions, achieves the objective of shortening or effacing the cervix, and opening or dilating it to at least 10 cm in diameter to allow the passage of the infant from the uterus to the vagina. It is comprised of two phases; latent and active.

There is compelling evidence that the duration of both latent and active phases of labor are clinically relevant and thus require consistent approaches to measurement. A prolonged latent phase of labor has been associated with an increased risk for oxytocin augmentation of labor, caesarean section, meconium staining in the amniotic fluid, 5-min Apgar score less than 7, need for newborn resuscitation and admission to the NICU [2, 3]. Women who are admitted to labor wards in the latent vs. active phase of labor are at higher risk for obstetrical intervention including electronic fetal monitoring, epidural analgesia, oxytocin, and caesarean section [4–7]. There may also be important differences in durations of latent and active phase labor and their relationship to obstetric outcomes according to parity.

Despite research pointing to the importance of the duration and transition between the latent and active phases of labor, there is considerable inconsistency in definitions of labor onset, a necessary component of measuring duration. The onset of the latent phase of labor has been defined as the time of the first clinical assessment in labor at the hospital [3, 5], or alternatively the beginning of strong regular painful contractions [2]. Similarly, inconsistency exists in definitions of the transition from the latent to the active phase. This important indicator of labor progress has been variably characterized as coinciding with the onset of regular contractions [8], beginning at the time at which the woman was admitted to the labor ward [9], when she seeks professional care [10], or the time at which she is consented for participation in a randomized controlled trial [11]. Recently researchers have used the woman’s self-report as the time of labor onset [8, 12–14].

Friedman originally defined the onset of the active phase of labor as the point in time when the rate of change of cervical dilatation significantly increases [15]. In practice many clinicians view 3 or 4 cm cervical dilation as the beginning of active phase labor [16], including the WHO’s partograph which is based on the principle that active phase of labor commences at 3 cm cervical dilatation and that during active labor the rate of cervical dilatation should not be slower than 1 cm/h [17]. Zhang et al.’s study of 1329 women in spontaneous labor at term with a singleton fetus in vertex presentation found contrasting findings. They reported that the cervix dilated substantially more slowly in the active phase than had been reported by Friedman, taking approximately 5.5 h to dilate from 4 cm to 10 cm, compared with Friedman’s reported 2.5 h and concluded that most women entered the active phase between 3 cm and 5 cm of cervical dilation [18]. A more recent retrospective study that analyzed labor trajectories of 62,415 women who vaginally delivered a singleton fetus with vertex presentation reported that the 95th percentile rate of active phase dilation was substantially slower than the standard rate derived from Friedman’s work, varying from 0.5 cm/h to 0.7 cm/h for nulliparous women and from 0.5 cm/h to 1.3 cm/h for multiparous women [19].

Influenced by this work, the American College of Obstetricians and Gynecologists recently released an obstetric care consensus statement explicitly stating that contemporary labor progresses at a rate substantially slower than historically believed. They state that because the maximal slope in the rate of change of cervical dilatation (i.e., the active phase of labor) did not start until at least 6 cm, a cervical dilatation of 6 cm should be considered the threshold for the active phase of most women in labor [20].

The controversy around definitions of labor onset probably stems, at least in part, from the lack of clear understanding of the biology of parturition. Changes in levels of fetal adrenal, pituitary, and placental hormones, paracrine signalling molecules and inflammatory mediators, occur on a continuum over a period of days to weeks and initiate factors that act to promote uterine activity [21], but none of these mechanisms have been completely elucidated [22, 23]. Consequently clinicians must rely on observable characteristics of labor to define its onset.

To clarify concepts surrounding the definition of onset of the latent and active phases of labor, and to determine what, if any, scientific rationale these definitions are based on, we performed a systematic review of the literature. Our review asks: 1) Among healthy women laboring spontaneously, how is the onset of the latent phase and the active phase of labor defined?; and 2) What, if any, evidentiary basis is provided by authors to support their definitions of labor onset?

## Methods

### Search methods

We searched for English, French or German-language original research papers published from 1978 to March 2014 that examined onset of the latent and active phases of the first stage of labor. The starting date of this search was chosen to reflect the publication date of the second and most recent edition of Friedman’s seminal book on the topic entitled “Labor: Clinical Evaluation and Management” [15]. We followed the PRISMA statement for reporting, although we declined to undertake risk of bias assessment as it was not pertinent to our research question, and no review protocol exists for this study.

We sought original research that defined or operationalized the onset of latent labor and/or active labor in a population of healthy women with term births. To focus on healthy women, we excluded studies that specifically focused on cohorts of women with health conditions in labor (e.g., women with gestational diabetes, gestational hypertension, or obesity). In order to identify appropriate studies an information specialist (DG) searched the following electronic databases: CINAHL, EMBASE, MEDLINE, the Web of Science, and Evidence-Based Medicine Reviews (which incorporates ACP Journal Club, Cochrane Central Register of Controlled Trials, Cochrane Database of Systematic Reviews, Cochrane Methodology Register, Database of Abstracts of Review of Effectiveness, Health Technology Assessment and NHS Economic Evaluation Database). We also traced citations to and from relevant articles, and searched our personal libraries for additional articles. As we were primarily interested in understanding how studies were defining the onset of labor, we searched databases using subject heading and key words clustered around the concepts of latent and active phase of labor onset or onset of the first stage of labor overall. See Appendix 1 for the full electronic search strategy for each database. No review protocol was published for this study.

### Study selection

We included studies of healthy women in uncomplicated labor at term written in English, French or German. In order to be eligible for inclusion, studies were required to be original, empirical research, and a study outcome must have involved labor onset or duration of labor. We excluded studies that focused exclusively on women with induced labor (although populations that included some women with induced labor were included), as well as case-studies, case-series and studies that did not present any original data (such as commentaries and reviews).

Papers were screened without blinding through a sequence of title (by SM and GH), and abstract (by SM and GH), and any discrepancies were resolved through discussion and agreement. If agreement could not be reached, a third screener (PJ) made the final decision [24]. A larger group conducted full text review (SM, GH, PJ, MG, HS, and VH). Each paper was reviewed by one of the original screeners (SM, GH and PJ) as well as a second screener (MG, HS and VH). Discrepancies were resolved by one of the original screeners (SM, GH and PJ) who had not read the full text of the article. Screeners did not screen or extract articles they had authored or coauthored [24].

### Data extraction and analysis

A standardized data extraction form was developed [24] to include details about the study design, setting, time period, and the inclusion and exclusion criteria used to define the study population, as well as information about the sample size, the intervention(s) of interest, and the outcome(s) of interest. Finally the reviewers independently extracted the definition of labor onset used according to whether it defined the onset of the latent, or active phase of labor or simply the onset of the first stage of labor. In addition, the reviewers extracted information about whether, and what, rationale the authors provided regarding their choice of definition of labor onset, including supporting citations.

Prior to beginning data extraction, all six full text reviewers independently piloted the standardized data extraction form on a random sample of three of the included studies [24]. Responses were compared for discrepancies and all reviewers were involved in revising the data extraction form to ensure consistency and improve data quality. Once the form was finalized, full text reviewers (SM, GH, PJ, MG, HS, and VH) independently extracted data from the studies. Each study was extracted by two reviewers including one of the original screeners (SM, GH, and PJ). We did not contact any study authors for data confirmation. As our primary interest was the definition of labor onset, rather than the validity of the conclusion or the study outcomes, we did not assess risk of bias in our included studies.

### Synthesis of results

We examined key aspects of the included studies, including study design, research objective, sample size, country of origin, years of data, and publication year, and constructed tables and figures to illustrate key findings. We also assessed differences in labor definitions according to parity.

## Results

### Description of included studies

**Fig. 1:**
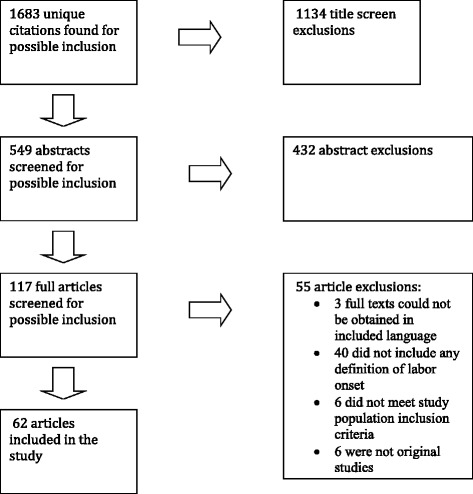
PRISMA/QUORUM diagram

**Fig. 2:**
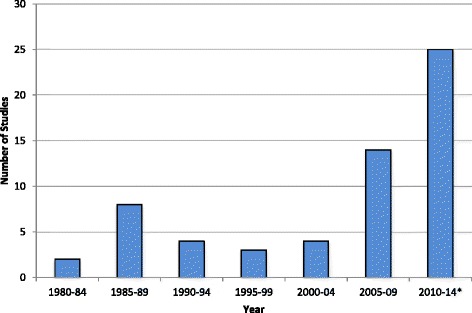
Frequency of included studies by publication year

We identified a total of 1683 potentially relevant citations (Fig. 1). Following title review, 549 were retained and review of these abstracts eliminated all but 117 studies. After full text screening, 62 studies were deemed eligible for inclusion in our review (see Table 1). Of the 62 included studies, 22 (35 %) were from the United States [25–44] and six (10 %) were from Germany [12–14, 45–48]. The remaining studies included four each from Italy [49–52] and Nigeria [53–56], three (5 %) each from Iran [57–59] and Norway [60–62], two each from Israel [63, 64] and South Africa [65, 66], and one each from Australia [67], Austria [68], Bahrain [69], Canada [70], France [45], India [71], Ireland [72], Jordan [73], Korea [74], Kuwait [75], New Zealand [76], Pakistan [77], Philippines [78], Saudi Arabia [79], South Korea [80], and Sweden [81]. Most of the included studies (*n* = 39, 63 %) were published between 2005–2013 (Fig. 2) [13, 14, 28, 30–34, 36, 38, 39, 42–48, 50–52, 55–59, 63, 67, 69–71, 73–77, 79–81]. The majority of studies were retrospective cohort studies (*n* = 29, 47 %) [2, 25, 27, 28, 30–36, 38, 40, 41, 43, 44, 49–51, 55, 61, 62, 65, 66, 70, 72, 80, 82, 83], while 29 % were prospective cohort studies (*n* = 18) [26, 29, 37, 39, 42, 46–48, 52–54, 56, 60, 67, 71, 74, 79, 81] and 11 % (*n* = 7) were randomized controlled trials or cohort [57–59, 69, 75, 77, 78]. The remaining eight studies (13 %) employed a range of qualitative, case control, mixed methods, or other research designs [12–14, 45, 63, 68, 73, 76]. Five studies (8 %) defined definitions of labor onset differently for nulliparous and multiparous women [36, 40, 41, 54, 66]. Of these five studies (8 %), four were published in 1986 or earlier [40, 41, 54, 66].

**Table 1:** Characteristics included in definitions of onset of labor

Characteristic included	*N* (%)
Type of labor defined	
Latent	3 (5)
First stage	11 (18)
Active	22 (35)
Labor (unspecified)	15 (24)
More than one of the above	11 (18)
Cervical dilation	42 (68)
In latent phase labor	
< 2 cm	1 (2)
3–4 cm	3 (5)
> 4 cm	7 (11)
In active labor	
≥ 2 cm	2 (3)
3–4 cm	10 (16)
> 4 cm	14 (23)
In first stage labor	
3–4 cm	4 (6)
> 4 cm	1 (2)
In unspecified labor	
≥ 2 cm	2 (3)
3–4 cm	2 (3)
> 4 cm	2 (3)
Cervical effacement	12 (19)
Regular painful contractions	44 (71)
Frequency of contractions	12 (19)
1 in 8–10 min	3 (5)
2 in 10 min or five minutes apart	3 (5)
3 in 10 min	5 (8)
1 every 3–5 mins	2 (3)
Other physiological symptoms	7 (11)
Rationale for definition	
Referred to women’s reports of onset or routine clinical practice	11 (18)
Cited Friedman	8 (13)
Cited Gross	4 (6)
Cited textbooks	3 (5)
Cited another study	2 (3)

1. How is the onset of labor onset defined?

### Types of labor onset defined by the included studies

We classified the type of labor onset according to what the authors of the included papers said they were defining. In the 62 included studies, we observed four distinct types of labor onset including “active labor”, “latent or early phase labor”, “first stage labor” or simply “labor” without further specification, which we call “unspecified labor”. The majority of studies defined the onset of active phase labor only (*n* = 22, 35 %) [26, 27, 31, 35, 41, 42, 45, 50, 51, 53, 56, 57, 59, 61, 63, 65, 66, 71, 74, 77, 79, 83]. Three studies only defined the latent phase of labor (5 %) [40, 55, 76], while 11 studies only defined onset of the first stage of labor (18 %) (see Table 1) [32–34, 38, 47, 62, 68–70, 73, 78]. Approximately one quarter of studies provided only a definition for unspecified labor (*n* = 15, 24 %) [12–14, 25, 30, 36, 37, 39, 43, 44, 46, 48, 49, 52, 58]. With respect to studies that defined more than one ‘type’ of labor, 10 studies (16 %) provided a definition for both active and latent phase of labor [2, 28, 29, 54, 60, 67, 72, 75, 81, 82], while one (1.6 %) defined both active labor and unspecified labor [80].

### Components of definitions of labor onset

Most studies (68 %) included measures of cervical dilation in their definition with only 20 studies omitting a specific measurement of dilatation from definitions of labor in their paper [12–14, 25, 29, 30, 32, 33, 35, 37, 40, 46–48, 61, 62, 70, 73, 76, 83] (Table 1). Regular painful contractions were also frequently referenced in definitions of onset of labor (71 %), with only 18 studies omitting mention of contractions [26, 27, 31, 36, 39, 41–45, 53, 55, 57, 59, 63, 68, 78, 79]. Studies also varied in their descriptions of the length and frequency of contractions at onset of labor. Twenty-one studies (34 %) included mention of either length or frequency of contractions in their definition of the onset of labor [29, 30, 32, 33, 35, 49–51, 54–56, 60–62, 66, 67, 69, 71, 74, 75, 83]. One study stated that onset of labor in general is also characterized by intact, rather than ruptured, membranes [28]. Below we outline how these commonly referenced components of labor definitions varied according to the type of labor defined.

### Latent phase onset

Among the 14 studies that defined latent phase labor, 11 (79 %) included cervical dilatation in the definition. Onset of the latent phase of labor was defined using various measures of cervical dilation, most commonly <4 cm (*n* = 7, 50 %) [2, 28, 54, 60, 75, 81, 82]; however, ≤2 cm, [72] and <3 cm [29, 55, 67] were also included in definitions. One study provided different definitions for the end of latent phase labor according to parity, indicating that a cervical dilation of 3 cm marked the end of the latent phase of labor for primiparous women, while for multiparous women it was 4 cm [54]. Cervical effacement was included in the definition of latent phase of labor in three of thirteen studies (23 %). While two stated that effacement should be at least 80 % [29, 75], the third study defined latent phase labor onset as when the cervix has “minimal or no effacement” [55].

All thirteen studies (100 %) that provided definitions for the onset of latent phase labor included the presence of regular painful contractions in their definition [2, 28, 29, 40, 54, 55, 60, 67, 72, 75, 76, 81, 82]. Three studies (23 %) stated that during the onset of the latent phase of labor there should be at least one painful uterine contraction every 8–10 min [29, 54, 55], and one study stated that there should be at least two painful uterine contractions every 10 min [75]. The duration of each contraction was not included in these definitions. Only three studies (23 %) included other physiological symptoms in their definitions. These included bloody show [29, 72, 76] and fluid loss [72, 76], as well as gastrointestinal symptoms or irregular (non-repetitive) pain [72, 76].

### Active labor

Of the studies that included a definition of the onset of active labor (*n* = 33), 27 (82 %) included cervical dilatation in their definition [2, 26–29, 31, 42, 45, 50, 51, 53, 54, 56, 57, 59, 60, 63, 65–67, 71, 74, 75, 77, 79–81]. Two (6 %) included ≥2 cm cervical dilation as the measure of labor onset [50, 51], ten (30 %) cited 3–4 cm [29, 45, 53, 54, 59, 65–67, 77, 79], while fourteen (45 %) included ≥4 cm cervical dilation in their definition of active labor onset [2, 26–28, 31, 42, 56, 57, 60, 63, 71, 74, 75, 80, 81]. Two studies (6 %) did not quantify the amount of dilation present at onset of active labor and stated rather that there should be contractions leading to “cervical change” [35, 83]. Four studies (12 %) characterized onset of active phase labor as the point at which the cervix begins to dilate >1 cm per hour [2, 41, 63, 79].

Cervical effacement was mentioned in six definitions (21 %) of onset of active labor [50, 51, 66, 72, 74, 81]. One study mentioned the cervix being generally effaced [81], one suggested that ≥75 % effacement was indicative of active labor [72], while three others considered the cutoff to be at least 80 % effaced [50, 51, 74], and finally one study referred to a “fully effaced” cervix [66].

Over half of the studies defining the onset of active labor included regular painful contractions in their definition (*n* = 20, 60 %) [2, 28, 29, 35, 50, 51, 54, 56, 60, 61, 65–67, 71, 72, 74, 77, 81–83]. Among the studies that defined onset of active phase labor, two indicated that contractions should be five minutes apart [66, 67], and two stated that there should be at least three contractions in ten minutes [71, 74], while two more suggested contractions should occur every 3–5 min [35, 83]. One study indicated that onset of active labor is characterized by contractions that are 20–25 s in length [71], while two studies (with the same first author) stated that contractions be >40 s long [50, 51]. Two studies included additional physiological symptoms in their definition of onset of active phase labor: fluid loss [72] and bloody show [29, 72].

### First stage labor onset

Of the 11 studies that defined onset of the first stage of labor without referring to a particular phase [32–34, 38, 47, 62, 68–70, 73, 78], five (45 %) provided a specific cervical dilatation in their definition, including four that defined first stage labor onset when the cervix was 3–4 cm dilated [34, 38, 68, 69] and one study that used a cervical dilatation of ≥4 cm [78]. Three studies did not quantify dilation but stated that at first stage labor onset there should be “cervical change” [32, 33, 70]. Only one study that defined first stage labor included effacement in its definition (9 %), and mentioned only that there should be demonstrable effacement and dilatation of the cervix in their definition of first stage labor [38].

Most studies that defined onset of the first stage of labor included regular painful contractions in their definition (*n* = 9, 82 %) [32–34, 38, 47, 62, 69, 70, 73]. Only one study referred to duration or frequency of contractions at onset of first stage labor and indicated that contractions should be >40 s long [69].

### Unspecified labor onset

Among the 16 studies that included a definition of labor that did not specify a phase or stage [12–14, 25, 30, 36, 37, 39, 43, 44, 46, 48, 49, 52, 58, 80], six (40 %) included a specific cervical dilatation in their definition. These six were evenly split between 2 cm [49, 52], 3–4 cm [43, 48], and >4 cm [36, 39]. Two studies included cervical effacement in their definition of onset of unspecified labor, stating that the cervix should be “partially” effaced [49] or ≥50 % effaced [52].

Twelve out of sixteen studies (75 %) that defined labor onset for an unspecified stage or phase of labor included regular painful contractions in their definition [12–14, 25, 30, 37, 46, 48, 49, 52, 58, 80]. Of these studies, four had the same first author [12–14, 46] and used a definition of onset of first stage labor that included multiple physiologic symptoms derived from a qualitative study on women’s experience of onset of labor at term [12]. Three studies diagnosed the onset of unspecified labor when one of the symptoms included contractions occurring at least three times in a ten-minute interval [30, 49, 52].

### Definitions according to parity

Five studies provided a definition of labor onset that differed according to parirty [36, 40, 41, 54, 66]. One study indicated that latent phase labor and active phase began when the woman’s cervix was 3 cm or 4 cm dilatation for primiparous and multiparous women respectively [54]. Another suggested that labor (unspecified) began at 4 and 5 cm cervical dilatation for nulliparous and multiparous women respectively [36]. Two studies by the same authors reported that cervical dilatation was expected to occur at different rates based on parity (1.2 cm/h for nullips vs. 1.5 cm/h for multips) [40, 41].

### Definition by caregiver vs. parturient

Most studies did not attribute diagnosis of labor to be in the domain of a specific type of caregiver (e.g., nurse, midwife, physician). Nineteen studies (31 %) indicated that the woman’s self-reported symptoms were used to diagnose onset of labor [12, 13, 25, 30, 32, 33, 35, 37, 40, 46–48, 66, 67, 70, 76, 80–82]. In seven studies (11 %) clinicians included in their definition that the onset of labor was the time at which the woman was admitted to hospital [14, 28, 38, 46, 47, 66, 73]. Three studies compared definitions between women and their caregivers [46, 47, 66].

### Temporal patterns

Over the study inclusion period (1978–2013; see Fig. 2), there were no temporal patterns observed regarding the types of labor onset defined by studies (i.e., latent vs active) or the measures of cervical dilation (i.e., 3 cm vs 4 cm) that studies used to define onset of labor. Rather, studies used heterogeneous definitions throughout the time period. However, the majority of the studies that defined labor onset differently for nulliparous versus multiparous women were published in 1986 or earlier [40, 41, 54, 66].

2) What, if any, evidentiary basis is provided by authors to support their definitions of labor onset?

The majority of studies did not provide any rationale for their definition of onset of labor (*n* = 37, 60 %) [12, 25, 31–34, 39, 43–45, 47, 50, 51, 53–59, 61–63, 65, 68–71, 73–78, 80, 81, 83]. Eleven described women’s reports or routine clinical practice as a rationale [26–28, 30, 35–37, 52, 60, 66, 67]. For instance, the authors of one study stated “we chose 4 cm as a commonly accepted changeover point” between the latent and active phases of labor [28].

Eight studies (13 %) cited publications that were written by Friedman or used his 1954 definition of the labor curve as their rationale [2, 29, 40–42, 72, 79, 82], however not all studies used the Friedman definition correctly. For example, only three of these studies mentioned rate of dilatation [2, 41, 79], which is considered an important component of Friedman’s labor curve [84]. Three studies cited obstetrical and obstetrical anaesthesiology textbooks [38, 49, 82], including a chapter in a maternal-fetal medicine text [85], an obstetric anesthesiology textbook [86], and two chapters from Williams’ Obstetrics [87]. Two studies [2, 72] cited clinical studies of length of labor [88, 89]. Finally, four German studies sharing a common author [13, 14, 46, 48] referenced the definition of onset of labor from a qualitative study they had previously authored [12] on women’s experiences of onset of labor at term.

## Discussion

This systematic review provides an overview of how labor onset for healthy women is defined in the research literature and summarizes the evidence being used to support these definitions. We found studies providing definitions for four different types of labor onset; latent phase, active phase, first stage and unspecified labor. All four definitions commonly referenced cervical dilatation, cervical effacement, and uterine contractions, with little mention of other physiologic indications, such as bloody show and gastrointestinal symptoms. Cervical dilatation and regular painful contractions were the most common indicators of labor onset, regardless of stage or phase. However, there was little consensus on the degree of dilatation or regularity of contractions, even within definitions for the same stage or phase. The majority of included studies (60 %) did not provide any evidentiary basis for their definition of labor onset. Among studies that did provide evidence for their definition, the most common was a citation of Friedman’s labor curve.

We report that there is considerable discrepancy in definitions of labor onset in the research literature. Even among studies referencing the same type of labor onset (e.g., active phase labor) and indication of labor onset, there was little consensus, with the exception that 100 % of definitions of latent phase labor referenced the presence of regular painful contractions. This lack of consistency may be driven in part by the lack of standardized documentation of labor onset in the patient’s medical record. The lack of consistent documentation may both contribute to and result from the lack of a standardized definition. This discrepancy in definitions is also not surprising given that the physiologic mechanisms that stimulate the transition of uterine muscle from quiescence to regular contractions occur over a period of time, and on multiple levels, none of which are observable, and none of which yield clear biologic markers which would permit a definitive diagnosis of labor onset. The process of parturition begins days or weeks prior to the onset of observable labor. Placental estrogens, relaxin, and prostglandins ‘soften’ the collagen fibers in the cervix and make it more distensible [90]. Under the influence of estrogen, prostaglandins and distension of uterine tissue, uterine tissue is prepared for labor through cell multiplication and hypertrophy. Uterotropins, including oxytocin, raise levels of intracellular calcium, which stimulates contractions. Oxytocin secreted by the fetus also is a major contributor to increasing oxytocin levels in uterine tissue [91]. Oxytocin receptors increase in numbers in uterine muscle under the influence of estradiol as term approaches. Also under the influence of estrogens, the number of gap junctions in muscles increase. Gap junctions are transcellular membrane channels, which allow ion exchange between cells to propagate an electrical signal and subsequent muscle contraction [90].

A definition of labor onset that uses both endocrine levels and observable signs and symptoms might provide a reliable and valid measure at some point in the future. In practical terms, what is needed is a point in time after which labor should not only be expected to continue among healthy women, but beyond which, failure to progress would require intervention on the part of the caregiver to prevent subsequent maternal and neonatal morbidity.

Studies in our review were more likely to focus on active phase of labor than latent phase labor, which is of concern given the adverse outcomes associated with early hospital admission in latent phase labor [2, 3, 30]. A strong consensus around the definition of onset of latent phase labor is needed to ensure comparability of research findings, and subsequently to guide clinical diagnosis and intervention. Understanding when the transition between the latent and active phases of labor takes places is essential for designing initiatives to assist women to remain out of hospital during latent phase labor [92].

Our review supports the notion that measurement of cervical dilatation is dominant in the discussion of determining labor onset and the transition from latent to active phases [76]. Thus it is perhaps not surprising that women present to hospital when not in labor, as they are generally unable to assess their own cervical dilatation. Previous research has illustrated that descriptions of labor onset and progression that rely on cervical dilatation do not provide women with the means to understand how far they have progressed in their labor [76]. While healthcare providers may feel relatively certain about their diagnosis when women arrive at hospital prior to active labor, they are then faced with making a management decision that incorporates not only their diagnostic judgment but also cues regarding how well the woman is coping, family expectations, and institutional requirements. These factors may contribute to admission in latent phase labor [93].

A consistent and measurable definition of labor onset for each phase and stage is essential in order to identify departures from normal labor trajectories and avoid misdiagnosis of the onset of labor with subsequent sequelae, including increased risk for oxytocin augmentation of labor, caesarean section, meconium staining in the amniotic fluid, 5-min Apgar score less than 7, need for newborn resuscitation and admission to the NICU [2, 3]. Definitions tend to be static, for example a measure of the cervical dilatation at which a phase or stage of labor is considered to have begun (e.g., active labor begins at 4 cm), or a degree of effacement. These static definitions may result from the widely held, and erroneous [84] conclusion that Friedman defined the transition from latent to active phase labor as occurring at 3–4 cm cervical dilatation [94, 95]. Friedman asserts instead that slow labor progression is identified by change in dilatation over time with active-phase cervical dilatation progressing linearly at a minimum of 1.0 cm/h in nulliparas [84]. Recent recommendations have changed the cervical dilatation upon which the transition is believed to take place to 6 cm [20]. Our systematic review has revealed that there appears to be little consensus in the amount of cervical dilatation necessary to indicate that active phase labor has begun.

Strengths of our systematic review include explicit, and detailed eligibility criteria and a comprehensive search constructed and conducted by an information specialist. We were also able to review studies published in English, French and German due to the multi-lingual capacity of our international team. A limitation of our review is that we cannot recommend a specific definition of labor. Given that our review sought simply to answer what definitions were in common use in the literature and what evidentiary basis was provided for their use, we were unable to assess whether specific definitions were associated with better obstetric outcomes than others. This is the type of research that will be needed to recommend a definition of labor onset. Further research seeking practitioners’ views on the most useful definition of onset of early labor would also be useful.

## Conclusion

In summary, we report very little consensus regarding definitions of labor onset in the research literature. In particular we note that latent phase onset is an understudied phenomenon whose definition merits further investigation by clinical scientists. Most definitions referred to the presence of regular uterine contractions and cervical dilatation as static concepts. Despite the fact that the current focus on static definitions of labor onset has failed to lead to consensus, recent recommendations continue to use this approach [20]. Future research could include testing definitions of labor onset that include other physiologic parameters such as station of the baby and measures of change in parameters over time. Given that Friedmans’ work seemed to be the most foundational in this body of literature, initial studies could compare definitions to the traditional Friedman model. Furthermore, emerging definitions need to be evaluated with respect to impact of their use on maternal and fetal outcomes, for example maternal pelvic floor injury, chorioamnionitis, hypoxic ischemic encephalopathy, and birth injury. While conducting this critical research, investigators would be well advised to keep in mind the balance between an objective and useful definition that will accurately indicate when interventions are warranted, and measures that can be used to help women self-diagnose labor onset and assist them in remaining out of the hospital during latent phase labor.

## Appendix 1

### Search strategy by database

Medline (Ovid MEDLINE(R) 1946 to Present with Daily Update)

Searched on 18 January 2013; updated on March 14^th^ 2014

1. Labor Onset/

2. Labor Stage, First/

3. 1 or 2

4. limit 3 to yr = “1978 -Current”

5. limit 4 to (english or french or german)

CINAHL with Full Text (Ebsco Host)

Searched on 23 January 2013

(MH “Labor Stage, First”)

Limiters: Exclude MEDLINE records; Language: English, French, German; Source Types: Academic Journals, Books, Dissertations, CEUs

Embase 1974 to 2013 January 22 (OvidSP)

Searched on 23 January 2013; updated on March 14^th^ 2014

1. labor onset/

2. labor stage 1/

3. 1 or 2

4. limit 3 to yr = “1978 -Current”

5. limit 4 to human

6. limit 5 to to (english or french or german)

7. limit 6 to exclude medline journals

Web of Knowledge (Thompson Reuters) Databases = SCI-EXPANDED, SSCI, A&HCI, CPCI-S, CPCI-SSH Timespan = All Years

Searched on 23 January 2013; updated on March 14^th^ 2014

1. Topic = (“labor onset” or “labor onset”)

2. Topic = (“Labor stage, first” or “Labor stage, first” or “labor stage I”)

3. #2 OR #1

4. Exclude Portuguese

EBM Reviews - Cochrane Central Register of Controlled Trials December 2012 (OvidSP)

Searched on 23 January 2013; updated on March 14^th^ 2014

1. Labor Onset/

2. Labor Stage, First/

3. 1 OR 2

4. limit 3 to yr = “1978 -Current”

(n.b. no language limits available; no MEDLINE or EMBASE records to eliminate)
